# Attitudes to aging mediate the relationship between older peoples’ subjective health and quality of life in 20 countries

**DOI:** 10.1186/1477-7525-11-146

**Published:** 2013-08-28

**Authors:** Gail Low, Anita E Molzahn, Donald Schopflocher

**Affiliations:** 1 Faculty of Nursing, University of Alberta, 11405-87 Avenue, Edmonton, AB T6G 1C9, Canada; 2 Faculty of Nursing, University of Alberta, Edmonton, AB, Canada; 3 School of Public Health and Faculty of Nursing, University of Alberta, Edmonton AB, Canada

**Keywords:** Attitudes to aging, Subjective health, Quality of life, Statistical models

## Abstract

**Background:**

With ever-increasing life expectancy globally, it is imperative to build knowledge of how older peoples’ views of their own aging, considering their health-related circumstances, affect quality of life for practitioners and policy-makers alike. Based on our literature review, we wanted to determine whether older adults’ attitudes toward their own aging would partly mediate the effect of their health satisfaction ratings upon their quality of life. Furthermore, would these attitudes mediate the relationship between health satisfaction and quality of life in the same way when we account for older adults’ country of origin, and their age and gender?

**Methods:**

This was a secondary analysis of cross-sectional survey data collected in 20 countries taking part in the 2003 WHOQOL-OLD Field study. The study sample consisted of 4593 adults whom were, on average, 72.10 years of age (range = 60 to 100 years of age); 42.8% were female. The WHOQOL-BREF measured quality of life and health satisfaction. The Attitudes to Aging Questionnaire measured participants’ attitudes toward physical change, psychosocial loss, and psychological growth. All items in both questionnaires were measured on a 5-point Likert scale. Questionnaire responses were analyzed using multilevel modeling and path analysis.

**Results:**

All three attitudes to aging partly mediated the relationship between health satisfaction and physical, psychological, social, environmental, and global quality of life. These partial mediations manifested in the same way across all 20 country samples, regardless of age or gender. Attitudes toward physical change were the strongest mediator of health satisfaction upon global and domain-specific quality of life, followed by psychosocial loss and psychosocial growth.

**Conclusions:**

Our study is the first cross-cultural study with a large sample to show that quality of life judgements, between 60 to 100 years of age, are a product of older men’s and women’s perceptions of health-related circumstances, and attitudes toward physical and psychosocial aspects of the aging self. A prospective study of the linkages between older peoples’ subjective views of health and attitudes toward the aging self over time using multiple subjective measures of health is warranted. Understanding these linkages may help practitioners and policy makers consider strategies to enhance quality of life.

## Background

From a developmental perspective, striving for a sense of integrity (versus despair) is one of the hallmark attributes of older adulthood; this is particularly challenging when one’s health-related circumstances are less favorable [1]. Poor health can interfere with older peoples’ integrity work or ability to accept changes in overall physical function and appearance, come to terms with losses of social roles, and foresee opportunities for growth in an unknown future [2,3]. In the quality of life (QOL) literature, little attention is given to attributes marking older adulthood [4] yet none of us are exempt from the developmental work associated with this life stage [3]. With ever-increasing life expectancy globally, factors affecting older peoples’ capacity to live a longer life of quality is a pressing issue [5,6]. Hence, it is imperative to build knowledge of how older peoples’ views of their own aging, considering their health-related circumstances, affect QOL for practitioners and policy-makers alike. One way to build such knowledge is to examine older peoples’ attitudes toward their own aging.

We understand attitudes to be stable integrative judgements that summarize the thoughts, feelings, and memories people have toward objects or situations from their direct experience or observation [7-9]. Attitudes can be formed about important life events [8] and attributes characteristic of those events [9], for example, the developmental stage of older adulthood. Thus, based on their observations of other older people an older person might judge that physical changes are part-and-parcel of getting older and may more positively evaluate their own aging in terms of physical changes. That is, attitudes towards aging may mediate self-reports of physical and psychological states. Across 20 country samples, we investigate how men’s and women’s attitudes toward their own aging in relation to physical change, psychosocial loss and psychological growth, and perceptions of their health-related circumstances affect their QOL between the ages of 60 to 100.

Various measures of health and functioning have been linked to attitudes to aging among older adults. Subjective perceptions of physical, cognitive and social aging have been found to influence mental health in terms of symptoms of depression, self-esteem and morale [10]. In other studies, age-related morale has explained respiratory-related mortality [11], longevity [12], and the capacity for activity in and outside of the home over time [13]. Poor health has had a negative impact on perceptions about old age and of aging in general [14]. Poor health may also influence how old a person feels versus how old they actually are [15]. Feeling older has also been associated with more pessimistic attitudes about aging as it relates to thinking, sharpness, and memory [16]. Being in good health has been found to reduce anticipation of physical and social loss [17], increase the tendency to frame older age as a time of continuous growth, lesser physical decline and social loss [18], and decrease negativity toward the future and physical change, including bodily aging [19,20]. Symptoms of depression have negatively affected attitudes toward psychosocial growth [21] as well as age identity, life satisfaction ratings, and affect [22]. Others have related higher ratings of mental and physical functioning to more positive age-related expectations about energy and pain, time with friends and family, and forgetfulness [23]. Being in good to excellent health also appears to be related to higher QOL [24].

Quality of life judgements are a product of the individual’s subjective view of illness and his or her view as to how this affects the physical, psychological and social self [25]. In other words, illness might be interpreted by individuals differently as detracting from or contributing to QOL based on their attitudes toward the aging self. In a psychometric study of the WHOQOL-BREF, significant correlations were reported between older peoples’ attitudes toward aging (specifically physical change, and psychosocial loss. and psychological growth), and their physical, psychological, social, and environmental QOL ratings [26]. Middle-aged adults’ actual (versus perceived) age has been found to predict their beliefs, over time, about the relative QOL in older adulthood compared to younger adulthood [27]. Other findings provide further evidence of a direct link between health and attitudes to aging; specifically, subjective ratings of health have been found to explain self-appraisals of older age through multiple health problems from midlife onward [28] and in older age [29]. Similarly, older people who appraise their health in a positive light have been found to report better age-related morale when one considers their will to live [12] and opportunities to continue achieving in life [13]. Believing that older age is a time of personal development [30] and better age-related morale [31] has been found to mediate the effect of subjective health ratings on life satisfaction. Attitudes toward psychosocial loss have been found to partly mediate the effects of perceived memory, mental status, and capacities for activities of daily living upon the QOL of older adults with dementia [32]. Hence, we hypothesized that older adults’ attitudes toward their own aging would mediate, at least in part, the effect of their health satisfaction ratings upon their global, physical, psychological, social, and environmental QOL.

Beliefs about the cultural institutions and norms (as a broad psychological factor) potentially influence how information is used to make QOL judgements [25]. Whether and how subjective views of illness affect aspects of the self could differ based on a person’s country of origin. This emphasis on culture is consistent with definitions of QOL as a culturally-sensitive appraisal [33] and of attitudes as part of cultural group membership, in that they originate from in-group norms and thus are an expression of one’s social identification with the group [34]. Country of origin has been found to influence age-related morale [35] and subjective age-identity [22] among adults of all ages, and perceptions of older age as a time of physical and social loss and change from midlife onward [21]. However, other studies of older adults across two countries have yielded evidence to the contrary [14,36]. Attitudes toward health have also significantly differed between older people residing in eight different European countries [37]. Hence, we asked: would older adults’ attitudes toward their own aging mediate the effect of their health satisfaction ratings upon their QOL in a different way when we account for which country they are from?

Age and gender have been found to consistently influence beliefs about the aging self in terms of age-related morale [11], aging in general [14], personal development [30], and physical and social loss [17]. Women appear to be at a disadvantage as they age, particularly when it comes to expectations of physical, mental and social decline [18,23]. Both age and gender have been found to also significantly shaped attitudes toward the physical changes of aging in Brazil, but when looking across cultures, age hardly influenced attitudes toward physical change and women were more positive about psychological growth [38]. The effect of gender on perceptions of bodily aging is less clear [14,36,39]. Subjective health appraisals can also be affected by age [13,26] and gender [13,28,40]. Hence, we also asked: would older adults’ attitudes toward their own aging mediate the effect of their health satisfaction ratings upon their QOL in a different way when we account for how old they are or whether they are a man versus a woman?

In summary, there has been little research relating to the linkages between attitudes to aging, health, and QOL. The findings from our literature review suggest that subjective ratings of health have explained self-appraisals of older age, and in two studies of older people, self-appraisals of older age have either been associated with or found to explain QOL. There is less literature regarding the influence of the older person’s country of origin, age, or gender. Hence we: (a) tested our hypothesis that older adults’ attitudes toward their own aging would partly mediate the effect of their health satisfaction ratings upon their global, physical, psychological, social, and environmental quality of life; and (b) explored whether these attitudes would mediate the relationship between health satisfaction and QOL in the same way when we account for older adults’ country of origin, and their age and gender. Understanding these relationships may help practitioners and policy makers consider strategies to enhance QOL.

## Methods

### Sample and procedure

We conducted a secondary analysis of data from the WHOQOL-OLD field study [41]. The purpose of the original study was to develop and test a new measure of QOL for older adults. Data were collected in 2003 simultaneously in 20 countries. These centres were: Melbourne, Australia; Paris, France; Geneva, Switzerland; Bath, England; Edinburgh, Scotland; Seattle, USA; Beer Sheeva, Israel; Barcelona, Spain; Tokyo, Japan; Izmir, Turkey; Vilnius, Lithuania, Prague, Czech Republic; Budapest, Hungary; Victoria, Canada; Oslo, Norway; Umea, Sweden; Copenhagen, Denmark; Leipzig, Germany; Porto Alegre, Brazil; Montevideo, Uruguay. Each centre in the WHOQOL-OLD field study received ethical approval and adhered to relevant local ethical standards. Data were collected on 5566 older people, primarily in community settings using a variety of culturally appropriate methods.

Each centre in the WHOQOL-OLD field study obtained their own IRB approval. This analysis is consistent with the original purpose of the WHOQOL-OLD field study.

### Measures

Participants’ perspectives of their own aging process were measured using the Attitudes to Aging Questionnaire or AAQ [42]. The AAQ contains 24 items representing three subscales: psychosocial loss, psychological growth, and physical change. The eight items in each subscale are measured on a 5-point Likert scale. Scores on the subscales range from 8 to 40. Item-response categories range from strongly disagree to strongly agree. On the AAQ, the higher a score is, the more positive the attitude towards one’s own aging process in that area. The subscale structure of the AAQ has been established in 15 countries using classical and modern psychometric methods [21,26,42]. The internal consistency reliability in this study was .86 for the whole instrument, and .81 for physical change, .74 and .81 for psychological growth and loss, respectively.

The WHOQOL-BREF is a short version of the WHOQOL-100 designed to measure generic QOL across cultures [43]. It has been translated into 50 languages. The scale contains four domains: physical (7 items), psychological (6 items), social relationships (3 items) and environmental (8 items). Each item is scored on a 5-point Likert scale in relation to the last two weeks, and higher scores indicate higher QOL. Cronbach’s alpha coefficient values for the WHOQOL-BREF physical, psychological, social and environmental domains were 0.86, 0.79, 0.63, and .83 respectively. We also calculated the BREF score as the sum of the BREF domains [44]. For subjective health, we used the BREF item “how satisfied are you with your health?” with five response categories ranging from “Very dissatisfied” to “Very satisfied”.

### Data analysis

We conducted multilevel regression analyses to predict the WHOQOL-BREF scale scores using satisfaction with health, the three AAQ subscales and age and gender as independent variables and country as a random effect. All study variables had fewer than 3% missing values, and data were deleted list-wise prior to our analyses. We had complete data for analysis on 4593 participants.

First, we examined whether the intercept in the regression equation varied by country of origin, and then we tested whether the slopes of the independent variables differed across the 20 country samples. We used the Mixed Model module of SPSS for multilevel models [45] to examine these models. In accordance with the findings of the multilevel regression analysis, we subjected the correlation matrix of study variables to a path analysis to determine if the three AAQ subscales acted as mediators between health satisfaction and QOL. For path models, we used Mplus software [46] and for explicit statistical tests for multiple mediators, SPSS [47].

## Results

### Participant characteristics

In Table 1, descriptive statistics for the model variables for each country of origin sample are presented. Across all 20 country samples, the mean age was 72.10 (se = 7.92 years) and 42.8% were female. Just under one-third of all participants (26.9%; n = 1235) had primary schooling, 18.8% (n = 863) completed high school, and 43.3% (n = 1988) reported some form of post-secondary education. Slightly more than half were married/partnered (56.2%; n = 2581); otherwise, 5.7% (n = 261) were single, 7.1% (n = 326) separated or divorced, and 29.7% (n = 1364) widowed. Slightly more than half (59.3%; n = 2723) reported having, on average, one or more chronic illnesses, and a large proportion lived in their own home either assisted by others (41.3%; n = 1896) or unsupported (38.2%; n = 1754). Few lived in residential care and nursing homes (6.9%; n = 316). Nearly two-thirds (n = 3063) were retired; 9.1% (n = 418) engaged in paid work and 2425 (52.8%) volunteered.

**Table 1:** Study variable descriptive statistics across 20 country samples

**Country**	**Gender**	**Age**	**Satisfaction with health**	**Psychosocial loss**	**Physical change**	**Psychosocial growth**	**BREF**	
	***Male***	***M***	***M***	***M***	***M***	***M***	***M***	***N***
		***(SE)***	***(SE)***	***(SE)***	***(SE)***	***(SE)***	***(SE)***	
Edinburgh	34%	77.35	3.7	30.39	25.23	26.34	277.85	97
		(1.07)	(.11)	(.51)	(.53)	(.50)	(5.51)	
Bath	38.2%	69.16	3.8	32.12	27.82	25.84	286.99	128
		(.61)	(.08)	(.41)	(.47)	(.45)	(3.98)	
Leipzig	54.8%	72.32	3.3	29.16	27.99	25.3	278.09	310
		(.49)	(.05)	(.31)	(.30)	(.23)	(2.84)	
Barcelona	44.5%	71.6	3.1	26.9	26.44	27.5	240.48	218
		(.49)	(.06)	(.34)	(.33)	(.29)	(3.62)	
Copenhagen	49.7%	71.19	3.8	30.15	28.79	27.95	297.66	298
		(.47)	(.05)	(.29)	(.32)	(.27)	(2.74)	
Paris	50%	78.3	2.8	25.08	21.41	22.69	235.93	86
		(.79)	(.10)	(.67)	(.60)	(.55)	(5.32)	
Prague	40.4%	71.18	3.1	25.73	23.89	26.16	245.91	309
		(.44)	(.05)	(.33)	(.31)	(.23)	(2.74)	
Budapest	34%	73.47	3.1	26.4	24.3	26.29	239.43	279
		(.53)	(.05)	(.36)	(.31)	(.26)	(3.19)	
Oslo	48.4%	74.22	3.7	29.91	27.15	28.24	280.84	254
		(.51)	(.06)	(.30)	(.34)	(.28)	(2.81)	
Victoria	46.0%	72.93	3.81	31.91	28.17	28.93	304.7	202
		(.60)	(.07)	(.36)	(.41)	(.31)	(3.73)	
Melbourne	41.7%	75.62	3.6	30.46	26.5	28.33	280.29	331
		(.39)	(.06)	(.31)	(.29)	(.22)	(2.86)	
Seattle	42.5%	71.69	3.6	31.68	27.21	29.76	298.97	268
		(.51)	(.06)	(.34)	(.34)	(.23)	(3.27)	
Beer-Sheva	32.6%	70.16	3.5	29.4	27.94	28.16	273.67	196
		(.54)	(.07)	(.43)	(.39)	(.36)	(3.88)	
Tokyo	47.9%	68.53	3.4	28.38	28.32	25.49	265.30	144
		(.50)	(.08)	(.45)	(.43)	(.34)	(4.23)	
Umea	48.6%	72.15	3.6	29.55	26.82	28.23	275.26	393
		(.41)	(.05)	(.27)	(.28)	(.23)	(2.33)	
Porto Alegre	32.6%	71.69	3.7	30.07	28.3	30.46	283.09	319
		(.43)	(.05)	(.32)	(.26)	(.20)	(2.66)	
Montevideo	26.6%	72.90	3.8	27.04	29.79	30.50	281.37	184
		(.65)	(.07)	(.48)	(.34)	(.28)	(3.8)	
Izmir	47.7%	70.92	2.9	24.22	22.4	26.84	225.13	327
		(.29)	(.06)	(.33)	(.33)	(.24)	(3.12)	
Geneva	46.7%	73.84	3.7	30.93	27.20	24.35	292.67	122
		(.64)	(.08)	(.49)	(.32)	(.47)	(4.26)	
Vilnius	32.3%	68.45	3.0	25.45	26.07	27.40	234.09	282
		(.38)	(.05)	(.34)	(.31)	(.23)	(2.9)	

### Multilevel regression analysis

In the multilevel regression analysis of the WHOQOL-BREF scale (Table 2), it was found that while allowing the intercept to vary across each country (a random intercept model) was a clear improvement over a model with a single fixed intercept, there was no improvement in the model when we allowed the slopes of the independent variables to also vary (a random slope and intercept model). This allowed us to form a partial correlation matrix among the QOL, attitudes toward aging, and satisfaction with health variables that removed the effects of the older person’s country of origin, and age and sex.

**Table 2:** Multilevel model for global WHOQOL-BREF

**Model**	**Model parameter**	**Parameter estimates (95% CI)**	**−2 Log likelihood**
Fixed slope, random Intercept	Fixed effects		43485
	Intercept	28.4 (14.4, 42.4)***	
	Health satisfaction	20.4 (19.2, 21.7)***	
	Physical change	2.7 (2.5, 2.9)***	
	Psychosocial loss	2.2 (2.0, 2.5)***	
	Psychosocial growth	1.2 (0.9, 1.4)***	
	Age	.03 (−0.1, 0.2)^a^	
	Sex	−1.1 (−3.1, 0.9)^a^	
	Variances		
	Residual	1076.9 (1032.8, 1122.9)***	
	Intercept	82.8 (41.2, 166.8)**	
Random slope & intercept	Variances		43470
	Residual	1065.2 (1020.9, 1111.4)***	
	Intercept	44.2 (3.6, 549.6)^a^	
	Health satisfaction	1.8 (0.2, 20.6)^a^	
	Physical change	0.2 (0.05, 0.5)^a^	
	Psychosocial loss	0.01 (0.00, 14.5)^a^	
	Psychosocial growth	0.05 (0.01, 0.4)^a^	

Age and sex slopes fixed to allow convergence in the random slope and intercept model. Fixed Effects are not shown as they differ only marginally from the fixed slope, random intercept model. *CI* confidence interval. ^a^ NS, ** p < .01, *** p < .001.

### Partial correlation matrix and path analysis

**Figure 1:**
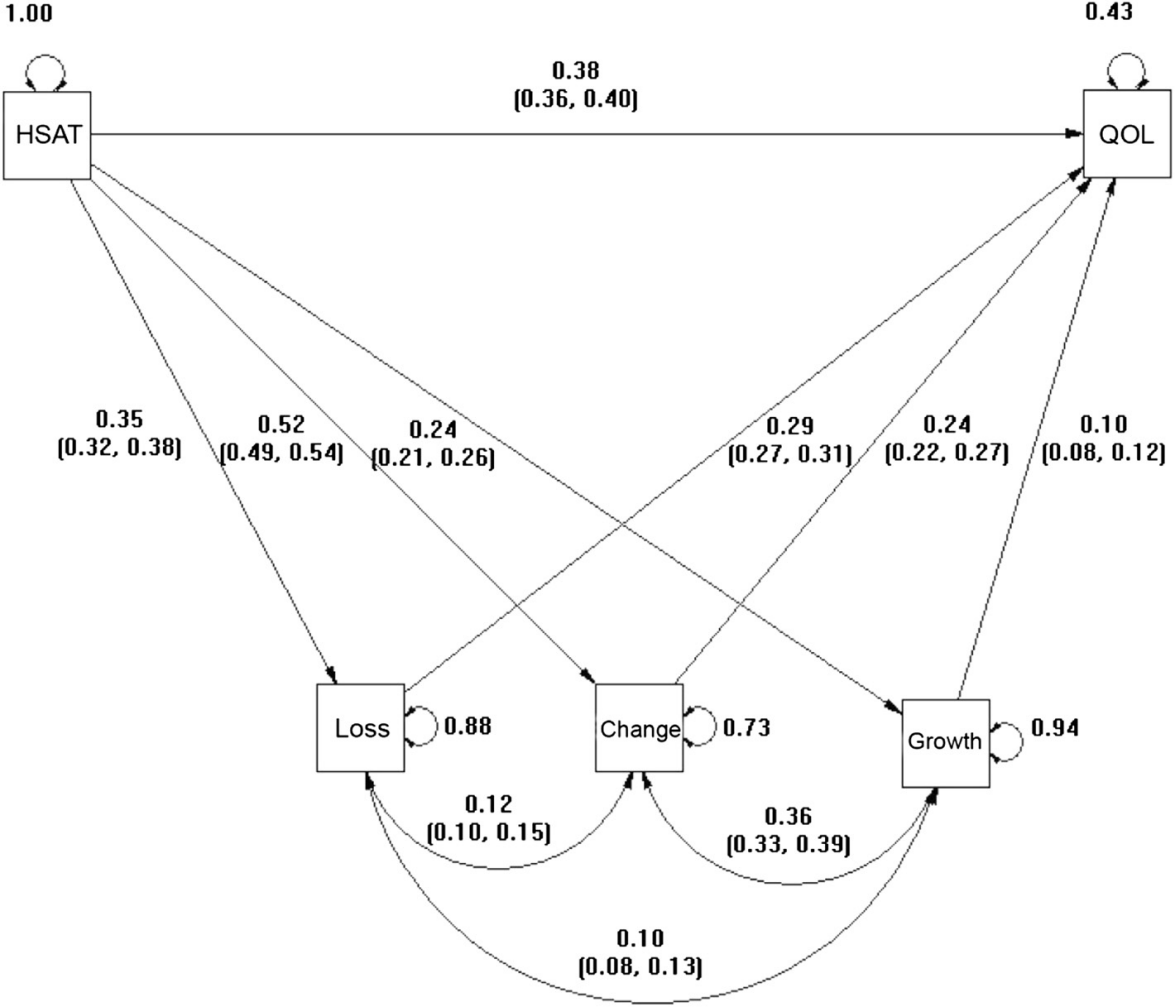
Global QOL path model with standardized coefficient and variance estimates.

Table 3 presents the partial correlation matrix^a^. Health satisfaction was significantly correlated with all three Attitudes to Aging subscales, and the global score on the WHOQOL-BREF. Correlations between the overall score of the WHOQOL-BREF and the attitudes to aging subscales were strongest for physical change and weakest for psychological growth. Figure 1 shows the standardized parameter estimates for the path model of this matrix [48]. The standard errors for the estimated beta weights were all statistically significant (p < 0.01) and ranged from se = 0.007 to se = 0.012.

**Table 3:** Relationships among health satisfaction, attitudes to aging scales, and WHOQOL-BREF

	**Satisfaction with health**	**Psychosocial loss**	**Physical change**	**Psychosocial growth**	**BREF**
Satisfaction with health	1.000	.429***	.545***	.274***	.669***
Psychosocial loss	.350***	1.000	.365***	.210***	.599***
Physical change	.515***	.303***	1.000	.481***	.607***
Psychological growth	.236***	.187***	.482***	1.000	.369***
BREF	.632***	.518***	.575***	.363***	1.000

Age, sex, and country partialled (below diagonal) and correlations (above diagonal). df = 4570. *** p < .001.

Testing for mediation involves estimating whether the path coefficient between health satisfaction and each mediator multiplied by the path coefficient between each mediator and the global BREF score is significantly different from 0. Bootstrap resampling was employed to estimate the confidence interval on the path products for each of the multiple mediators simultaneously [45,47]. The total mediation effect and each of the mediations through the Attitudes to Aging subscales proved to be statistically significant (all p values < 0.01). As can be seen in Figure 1, while health satisfaction directly affected QOL (beta = .38, p < .01), its effect on QOL was also partly mediated by all three Attitudes to Aging subscales. Path products for these mediations were highest for physical change (beta = .122, p < .01) and psychosocial loss (beta = .102, p < .01) compared to psychological growth (beta = .024, p < .01). The total effect of health satisfaction on QOL (which is identically the beta coefficient of health satisfaction in a model where the AAQ subscales are not included) was strong (beta = .628, p < .01).

Table 4 shows the parameter estimates for our model with the four specific domains of the BREF as dependent variables. Excepting the non-significant path from growth to environmental QOL, the level of significance and the direction of path relationships were identical to our global QOL model. While there are differences in path coefficients from attitudes to aging and each of the BREF domains compared to the model using global QOL, the means of the four domain-specific path coefficients for loss, change, and growth to each BREF domain were similar to their global QOL counterparts, partly due to the appreciable correlations among the BREF domains. These correlations ranged from .369 to .619 (p < .001).

**Table 4:** Parameter estimates for mediation models for WHOQOL-BREF Domains

**Model path**	**Physical**	**Psychological**	**Social relationships**	**Environment**
	**Parameter (95% CI)**	**Parameter (95% CI)**	**Parameter (95% CI)**	**Parameter (95% CI)**
Health satisfaction- > QOL	0.48(0.45, 0.50)**	0.28(0.26, 0.31)**	0.21(0.18, 0.24)**	0.24(0.21,0.27)**
Psychosocial loss - > QOL	0.22(0.20, 0.24)**	0.34(0.32, 0.36)**	0.26(0.23, 0.29)**	0.28(0.25, 0.30)**
Physical change - > QOL	0.31(0.29, 0.33)**	0.18(0.15, 0.20)**	0.08(0.05, 0.12)**	0.22(0.19, 0.25)**
Psychosocial growth - > QOL	−0.07(−0.05, -0.09)**	0.18(0.15, 0.20)**	0.15(0.13, 0.18)**	0.03(−0.01, 0.05)^a^

Remaining parameters to those presented in Figure 1. *QOL* quality of life, *CI* confidence interval. ^a^NS. ** p < .01

## Discussion

In this cross-sectional study of 4593 older people, attitudes toward physical change, psychosocial loss, and psychological growth partly mediated the impact of health satisfaction on QOL, and did so in the same way across all 20 country samples and among men and women at any age. This pattern of findings suggests that QOL judgments were significantly affected by participants’ satisfaction with health and attitudes to physical and psychosocial aspects of the aging self.

Participants who were dissatisfied with their health had more negative attitudes toward their own aging in terms of physical change. This finding is consistent with other studies of older people where it has been found that declining health can result in anticipation of future changes for the worse [19] and negative feelings regarding bodily aging [20]. Perceived health has also been most strongly associated with physical decline [18]. Being in good health in older age has been found to yield a sense of hope for maintaining health-related resources and physical well-being [17] rather than seeing oneself as getting old (in terms of physical health difficulties and concerns) [49], and change for the worse, including one’s energy [13]. Further support for these findings is our observation that attitudes toward physical change had the largest effect upon on our participants’ physical QOL in terms of their energy, sleep, mobility, and activities.

Participants’ health satisfaction ratings also affected their attitudes towards psychosocial loss. Benefits of positive perceived health include less anticipation of losses in social relationships and loneliness [17], and greater social interaction and involvement with volunteer and organized groups [50]. Social participation offers the opportunity to strengthen peoples’ sense of identity and belonging [51]. Thus, in terms of the AAQ subscales, the paths from psychosocial loss to psychological (positive feelings, thinking, esteem and body) and social (sex, support, and relationships) QOL in our study are consistent with other findings. The smaller correlations between loss and psychological and social QOL reported by others [26] do not account for the effects of satisfaction with health.

Health satisfaction had the weakest effect upon psychological growth, and this effect was negative. Nevertheless, this path coefficient from health satisfaction to psychological growth was significant across all 20 country samples. Perhaps lower health satisfaction is an impetus for older adults to explore opportunities for psychological growth. Poor health has been conceived of as a factor spurring on the search for meaning and purpose in life in older age [52]. Others argue that ill health is not synonymous with believing there is little left to achieve in older age [6]. Psychological growth had a modest effect on global QOL but its significance speaks to the importance of growth-enhancing generative activities such as sharing past accomplishments, receiving recognition in life, and planning future activities to older peoples’ overall QOL ratings [53].

Adaptation to physical and psychological change in later life can be expressed through being and doing in familiar environments, practicing familiar skills, and interacting with familiar social ties [54]. In this study, attitudes toward psychosocial loss and physical change had a similar significant impact on environmental QOL across all 20 country samples, and regardless of age or gender. Others have reported moderate correlations between attitudes toward loss and change in older age and environmental QOL across two countries but did not account for age or gender [26]. Accessible and quality environments provide opportunities for social interaction and support the realization of personal goals and projects [55]. Supportive environments have also enhanced older peoples’ sense of membership to a community [56] and place belonging [57]. Perhaps older peoples’ attitudes about psychosocial loss and physical change are an extension of environmental resources, incentives and constraints that facilitate adjustment and accomplishment in later life [58].

Of particular interest to us was that physical change had an impact upon social QOL that was two-thirds smaller than that of psychosocial loss. Far larger path coefficients have been reported over time between positive age-related morale, including physical energy, and perceived levels of social support among women with multiple sclerosis at a wide variety of ages [59]. Lesser age-related expectancies of physical decline among older Korean men and women have also been strongly associated, in part, with taking more responsibility for interpersonal relationships [60]. Others believe the importance of physical appearance in older age is rooted in a social standard of aging that is more forgiving of men and orientates women toward a youthful appearance [61]. However, women are not necessarily more negative toward physical signs of aging [14,36,39]. In our study, physical change appeared to be of equal social significance to men and women, and appeared to have a small impact on the quality of their personal and sexual relationships and anticipations of social support. Perhaps in our case, this small impact may be partly owing to older adults selectively investing their time and energy with people when they feel a more meaningful or closer connection [62]. Perhaps the personal relationships our participants continued to invest offered them unconditional physical regard. Relationships of older adults with people with whom they have affectionate ties have tended to depend less on what older people can do in terms of their physical performance; thus, even when physical declines are considerable, older people can still find intimacy in close relationships [63]. Late-life friendships, akin to sibling ties, are often characterized by a similar status in terms of age and social class, long-term reciprocity, and a shared history fostering self-continuity [64]. Similarly aged peers could also have intimate knowledge of what it is like to be living with the physical changes of aging [42].

The limitations of this study relate to a non-representative sample and use of an existing data set. Convenience samples were used to collect data in many of the countries, thus, our study sample is not likely to be representative of older people in the 20 countries. The sample is skewed to a healthier younger old population, given the mean age of 72 years of our respondents and that they were satisfied with their health. Hence, our findings may not apply to frail or older-old populations. Further, data were not available from all regions of the world. These limitations preclude generalization to other samples. The cross-sectional nature of our data prevented us from ascertaining causal relationships. The low reliability of the social domain of the WHOQOL-BREF is an added concern, and similar reliability scores have been noted in other studies [24,65]. Future research could explore the effect of socioeconomic status on these variables, since it has previously been found to influence attitudes to aging [11,15,28], QOL [66,67], and self-rated health [13]. For this study, a suitable measure of socioeconomic status was not available.

## Conclusions

In this study, older peoples’ attitudes toward their own aging with respect to physical change, psychosocial loss, and psychological growth partly mediated the relationship between their health satisfaction and QOL. These partly mediated effects manifested in the same way across 20 country samples, regardless of the age or gender of our 4593 participants. Participants’ attitudes toward physical change were the strongest mediator of health satisfaction upon global and domain-specific quality of life, followed by psychosocial loss and psychosocial growth. The direction of these partly mediated effects indicated that participants who were dissatisfied with their health harboured more negative attitudes toward physical change and psychosocial loss. In contrast, health dissatisfaction appeared to serve as an impetus for psychological growth. The subsequent effects of participants’ attitudes upon QOL, in some instances, were marginal; physical change in relation to social QOL serves as a case in point. Nonetheless all three attitudes were found to significantly impact participant QOL, both globally and across four life domains.

Our study is the first cross-cultural study with a large sample to show that QOL judgements between 60 to 100 years of age are a product of men’s and women’s perceptions of health-related circumstances, and attitudes toward physical and psychosocial aspects of the aging self. The findings of this study make a significant contribution to the QOL literature by shedding some light on the linkages between attitudes to aging, health, and QOL in older age, particularly across cultures. With ever-increasing life expectancy globally, factors affecting older peoples’ capacity to live a longer life of quality is a pressing issue. More research is essential. A prospective study that involves longitudinal data would certainly enhance our ability to make causal inferences. In this prospective study we would use multiple measures of subjective health to determine whether the relationship between health satisfaction and psychological growth can be replicated beyond this studied sample. Further research regarding the specific roles that friends versus family or intimate ties play in helping older people navigate the physical changes of aging and the effects of socioeconomic status would be important. To our knowledge, interventions for enhancing how older people view aging in relation to QOL have yet to be identified. Nevertheless, exploring how psychosocial interventions such as counselling, support groups, and socialization activities might affect perceived health and attitudes to aging could be valuable since these factors have a significant influence on QOL of older adults.

## Endnote

^a^The partial correlation matrix for WHOQOL-BREF domains is available from GL upon request.

## Abbreviations

QOL: Quality of life; Se: Standard error; CI: Confidence interval; HSAT: Health satisfaction.

## Competing interests

Neither the primary author nor the co-authors have any competing interests to report. This manuscript has been submitted for review to Health and Quality of Life Outcomes alone.

## Authors’ contributions

All authors contributed to the conception and design of the paper. AM was the primary investigator for Canada in the WHOQOL-OLD field study. DS conducted all data analyses. GL developed a draft of the manuscript. All authors took part in interpreting the data and making critical revisions. All authors read and approved the final manuscript.
